# Data-driven demand analysis and design reliability study of critical components of complex products

**DOI:** 10.1371/journal.pone.0319475

**Published:** 2025-03-24

**Authors:** Zhan Meng, Zhenggang Wang, Dapeng Gao, Wei Lu

**Affiliations:** 1 Henan Academy of Sciences, Zhengzhou, Henan, China; 2 School of Management, Zhengzhou University, Zhengzhou, Henan, China; 3 Logistics Management Section, SAIC Motor Passenger Vehicle Company (Zhengzhou Branch), Zhengzhou, Henan, China; 4 The First Affiliated Hospital of Zhengzhou University, Zhengzhou, Henan, China; 5 Institute for Hospital Management of Henan Province, Zhengzhou, Henan, China; Gazi University Faculty of Engineering: Gazi Universitesi Muhendislik Fakultesi, TÜRKIYE

## Abstract

Distinguished from the traditional general framework of product design, under the background of digital intelligence, the complex product design method presents the characteristics of rapid iteration and high degree of integration, and adopts the simulation and modeling technology, advanced production methods, etc. to further improve the quality and reliability of complex product design. Therefore, the demand analysis and design reliability study of critical components of complex products need to consider the sustainability, resources and economy of design, manufacturing and service. In this paper, starting from the product requirement analysis, the Kano-QFD model is used to clarify the requirement identification path of the critical components of complex products and ensure that the quality characteristics of the critical components of complex products are consistent with the customer requirements. The multi-dimensional characteristics of product design parameters, manufacturing process and quality are integrated to construct a design manufacturing analysis (DMA) model, which it’s based design reliability analysis method for critical components of complex products. Finally, the method proposed in this article was validated by taking the demand analysis and design reliability of critical components of automotive engines as an example. The study shows that the method proposed in this paper is highly compatible with the practice of complex product design and manufacturing at the early stage, and the method proposed in this paper also can provide product design and quality management reference for the research and development and manufacturing personnel of complex products to meet the changing needs and challenges of complex products.

## 1 Introduction

Rapid response to market demands and changes becomes the key to high quality and sustainable development of an organization, especially for complex products [1]. With the development of the Internet, a new generation of information technology, intelligent manufacturing and other key technologies, manufacturers of large and complex products are gradually changing the product development model. The traditional mode of demand analysis, conceptual design, detailed design, manufacturing and production, assembly and verification, production delivery and after-sales service are gradually transformed into a collaborative and integrated development of the whole life cycle of design, manufacturing and service. It is mainly embodiment is to concentrate advantageous resources to break through the critical components that affect the quality and reliability of complex products [2]. Complex product requirements analysis and design reliability research are interrelated and progressive processes. Through mutual support and feedback, they can effectively improve product reliability and meet user needs. In the product development process, requirements analysis is the first step, which plays a key role in determining product function, performance, manufacturing process, reliability requirements and cost. Reliability research is built on the basis of product requirement analysis, aiming to assess the reliability and failure rate of the product during the stages of use, manufacturing and operation and maintenance services, etc. Through reliability research, the failure mode, failure rate and life prediction of the product can be analyzed, and the reliability and life of the product can be effectively improved. In the requirements analysis stage, formulating clear reliability requirements can helps determine the design requirements of the product, while reliability research provides feedback and improvement directions for the requirements analysis by evaluating and verifying the reliability of the complex product. Quality design is an important part of large-scale complex product development, product reliability, especially the reliability of complex products in the era of digital intelligence is first designed, followed by advanced production and manufacturing technologies, such as intelligent manufacturing, digital twins and industrial Internet production. The contribution to product reliability, sometimes reliability design reaches 80%, manufacturing only 20% [3] and through a series of management tools to achieve the overall function of reliable, stable performance, high reliability products.

The emergence of a new generation of information technology has led to the further development of the collection, storage, analysis and application of complex product reliability data. Complex products in the design, manufacturing and operation and maintenance of the whole life cycle to produce a large amount of data and information, these data and conditions are called reliability data, is usually used to assess the complex products, systems or components in a particular condition of normal operation ability. For example, mean time between failures (MTBF), failure rate, availability, repair time, etc. are usually used to determine the product reliability metrics. These reliability data are essential for complex product design, manufacturing, operation and maintenance, and continuous improvement. They can effectively help to determine the life, performance and availability of the product in order to meet the user’s needs and improve the quality and reliability of complex products. Product demand analysis can be achieved by distributing research questionnaires, professional forum websites, after-sales service personnel, product design engineers, manufacturing engineers, quality management engineers and quality data of the product manufacturing process. And further feedback can be given to the design and manufacturing process to achieve iterative optimization of product design and customer requirements [4]. Design reliability is an important part of complex product development. Considering customer needs and manufacturing operability is the key to measuring the success of the design, and is also a key measure to achieve design reliability. The involvement of customers and suppliers helps overcome design limitations caused by complex structural and technical barriers and form optimal quality solutions.

Overall, the difficulties in demand analysis and reliability study of complex products are as follows:

1) Customer demands change rapidly and are highly uncertain. In the process of complex product development, its cycle usually takes 3–5 years. Due to market competition, technological innovation, customer feedback, etc. will cause changes in demand, and customers, research and development personnel, manufacturing personnel and other multi-participants will often bring the conflict of demand, design conflicts, which makes the product quality program deviates from customer demand.
2) Difficulty in collecting and managing customer requirements. With uncertain information and poor data, it is difficult to effectively collect requirements from various stakeholder groups (e.g., customers, market, research and development (R&D) staff, manufacturing staff, operation and maintenance staff, and competitors, etc.), and how to manage, record, and track these requirements effectively.
3) Lack of reliability data. In reliability research, a large amount of data is needed to evaluate the reliability indicators of a product or system. For new products and systems, there is a lack of sufficient historical data, which further affects the reliability evaluation work. In addition, different products have various failure modes and failure mechanisms at different stages. Identifying these complex failure modes and developing corresponding reliability testing and analysis methods have become research challenges.

Literature studies have shown that demand analysis of critical components of complex products [5] and their design reliability [6,7] studies have attracted a lot of attention from researchers. Requirement’s analysis and reliability studies of complex products require a high degree of technical knowledge, organizational skills and problem-solving skills. Dealing with these difficulties effectively can help ensure that products meet high quality standards and customer expectations. In this paper, the following issues will be investigated on this basis:

1) How to effectively identify customer needs and achieve effective transformation of customer needs and technical initiatives.
2) In the case of multi-dimensional coupling of design and manufacturing data, construct a design reliability research method for critical components of complex products.

In order to solve the above problems, this paper proposes to combine the Kano-QFD method to propose a demand identification path for critical components of complex products, which ensures that the quality characteristics of critical components of complex products are consistent with customer demands. Secondly, multi-dimensional features such as product design parameters and manufacturing are integrated to construct a DMA-based reliability research method for the design of critical components of complex products. Finally, the proposed method is validated by taking the demand analysis and design reliability of critical components of automotive engines as an example.

The paper is organized as follows: section 2 presents the literature review; section 3 focuses on the proposed methodology; section 4 gives an illustrative example, in which the results are also discussed; and finally, section 5 presents insights and suggestions for future research.

## 2 Literature review

In this section, we review research related to requirements analysis and design reliability of critical components of complex products.

### 2.1 Demand analysis of critical components for complex products

Rapid technological change, increasing product complexity, and relatively short marketing time challenges are the challenges faced by today’s manufacturing industry. Users want high quality and reliable products, and companies need to develop and launch new products that meet the needs of the users to maintain business continuity and win the competition for the manufacturing company [7]. The Kano model is widely used as a useful tool to understand customer needs and to analyze the impact of meeting customer needs on the level of customer satisfaction. The QFD model is used to translate consumer needs and desires and helps identify needs that affect customer satisfaction. The QFD method helps convert customer needs into technical requirements so that designers can decide the priorities for improvements or new product development. Thereby developing products that meet product and service design requirements [8].

Combining requirements analysis and quality function development to carry out requirements analysis of critical components of complex products has attracted increasing attention from researchers and practitioners, and valuable studies has emerged. Kim et al. investigated the development methodology of smart medical devices using the Kano-QFD model [9]. He et al. optimized the elasticity of the enterprise supply chain [10], Li et al. designed the innovation of smart home products for the elderly [11], Lyu et al. optimized the design of wooden desks in open-ended offices [12], Liang et al. based on the fundamental connection between key components introduces an analysis method for thermal networks based on matrix perspective technology and a thermal network for a typical Hermetically Sealed Electromagnetic Relay is designed [13], Hao et al. proposed a method for obtaining user requirements for complex products based on data mining, which is mainly used in the innovative design stage of complex products to solve the problem of effectively utilizing a large amount of design data [14].

More and more scholars are emphasizing the research on the correlation between customer needs and complex product quality and reliability, which is complicated to assess due to the complexity of influencing factors involved in complex product quality and reliability (e.g., service life, use in extreme space and environments, size, weight, cost, and complexity of maintenance, etc.). Wu et al. proposed a user demand-oriented baby stroller design solution centered on seeking the best function combination by combining Kano, QFD, and Function Analysis System Technique (FAST) methods by establishing a correlation between user demand and product function, and the results showed that the proposed feature combination design approach enables designers to effectively capture the real user needs and thus produce better designs to meet user expectations [15]. Temponi et al. developed a fuzzy logic-based quality house extension approach for capturing imprecise requirements to facilitate communication between design engineering, manufacturing engineering, marketing personnel, and other interdisciplinary teams of personnel and provide a formal representation of the requirements with a heuristic reasoning scheme to reason about the implicit relationships between requirements [16]. Chen et al. constructed an integrated QFD and failure mode and effect analysis (FMEA) approach for identifying critical components of complex products by considering key data such as customer requirements, failure causality between failure modes, importance correlation between risk factors, and customer demand for product components [17]. This method characterizes customer needs under fuzzy evaluation semantics by establishing quality function deployment, and uses directed networks to characterize product components and internal failure causal relationships. Aiming at the situation that the existing QFD customer requirements and their weight analysis have more subjective factors, resulting in less objective analysis results, Hu et al. proposed a QFD customer requirements mining method based on the data-driven of product reviews in terms of the theme extraction, the normalized expression of the requirements and the improvement of the proportional importance [18].

However, most of the existing research that combines the Kano-QFD model to analyze product design and innovation only studies one stage, and loses the analysis of subsequent stages of product design, manufacturing and innovation [19]. In the stage of complex product innovation design, a large amount of design data has not been effectively used, and there are some problems of low efficiency and lack of objectivity of user survey [13]. At the same time, there are relatively few studies on demand analysis for large and complex products due to the rapid changes in demand, high uncertainty, and difficulties in demand collection and management. In addition, there are few clear processes and methods to guide manufacturing companies on how to realize the requirements analysis and design process of complex products in an orderly manner.

### 2.2 Research on the design reliability of key components of complex products

In the fast-paced product development process, design reliability is critical to ensure product reliability while maintaining short iteration times. Product design begins with the conceptual design phase, which explores and defines the key functional features and components of the product. Although design details cannot be specified at this stage, this phase determines the overall product framework, which has a significant impact on the key product characteristics such as performance, reliability, and cost [6]. Differences in the number and intensity of physical relationships between the constituent parts of complex products. Complex products have the typical community structure characteristics, in a complex product, there are always some key components that affect the reliability of the entire complex product. Identifying the key components of a complex product and adopting targeted measures for them can effectively improve the reliability and service life of the product [20]. The key components of complex products are characterized by multi-level, multi-attribute, creativity and complexity, etc. In order to improve the efficiency and reliability of the products, methods such as QFD, Theory of the Solution of Inventive Problems (TRIZ), Taguchi’s Method, Failure Mode, Effects and Criticality Analysis (FMECA), Highly Accelerated Life Testing (HALT), load-strength analysis (LSA), etc. have been used in the R&D and design stage of the complex products.

Reliability is a fundamental requirement for products, especially in the industrial field. With emerging technologies, product complexity, and component miniaturization, the number and type of failures are increasing exponentially, which is the reason why design engineers need to focus their attention on functionality, performance, reliability, availability, maintainability, and safety, among other things. And it is well known that the right way to consider reliability, availability, maintainability, and safety is in the product design phase [21]. The purpose of designing for reliability is to eliminate failures of critical system functions in a system, and should concentrate on critical and major failures and complexities during the design process of complex products [22].

In product design, the initial design phase is receiving more and more attention because it has a significant impact on subsequent phases of product development, manufacturing, and operations and maintenance services. However, for larger and more complex products, it is very difficult to accurately predict product reliability during the initial design phase. Since the activities of designing complex products and systems (CoPSs) are mainly focused on redesigning existing CoPSs to meet customer needs and improve their reliability, identifying the components to be improved plays a key role in the redesign process. However, existing methods for improving the design of complex product components mainly consider customer needs and often ignore key information such as complex product failures and quality management to improve product reliability [23]. Liao et al. elaborated the definition and evolution mechanism of key reliability characteristic (KRC) of a product based on the symptoms of reliability degradation during the product life cycle [24]. They used association rule mining to determine the importance of parameters in the KRC association tree. The performance is sorted, and a KRC-driven manufacturing process reliability active control method and strategy using exponentially weighted moving average control chart is proposed. Catelani et al. believes that product redesign strategies can be effective in reducing design lead times and lowering manufacturing costs for innovation development [21]. The identification of critical functional components is the foundation of product redesign and is essential for improving product reliability. The use of component reliability importance to improve complex system reliability can be considered as a measure of the impact of each component on the reliability of the whole system. Taking into account reliability importance assessment during the design phase, engineers can optimize their efforts to improve system reliability by focusing on components that have the greatest impact on the overall system. Designers can identify system deficiencies based on reliability importance results and compare different solutions to enhance system robustness and reduce improvement time. In addition, Öner et al. proposed a decision support model considering maintenance cost, downtime cost, and spare parts inventory levels [25]. The decision support model can support the system to make decisions on the reliability level of key components during the design stage. Chen et al. proposed a component importance measurement method with continuous time degradation, and developed Copula Hierarchical Bayesian Network (CHBN) to perform system reliability estimation [26]. CHBN provides a decision support tool for designers to identify vulnerable components and ensure the overall reliability of mechatronic systems in the early design of the system. Existing assembly accuracy prediction methods rarely involve the multidimensional error coupling of parts and the influencing factors in the assembly process, Yi et al. presents a digital twin (DT)-driven assembly accuracy prediction method for the HPPA of complex products [27]. Zhang et al. proposes a SysML-based multidisciplinary reliability design optimization modeling method, enhancing the efficiency of design optimization for complex systems [28]. Hong et al. proposed a new reliability topology optimization method based on the reliability-and-optimization decoupled model and teaching-learning-based optimization (TLBO) algorithm [29].

Other studies have also tried to combine conceptual design with product reliability analysis to a certain extent, but most of them are limited to the identification of problems in the design stage [6,30–37].

In order to improve the reliability of critical components of complex products, this study starts from the product requirement analysis and adopts the Kano-QFD model to clarify the requirement identification path of critical components of complex products to ensure that the quality characteristics of critical components of complex products are consistent with the customer requirements. On this basis, multi-dimensional features such as product design parameters and manufacturing are integrated to construct a DMA-based reliability research method for the design of key components of complex products. In order to verify the applicability of this paper, the engine and cylinder block are taken as examples for research.

## 3 qResearch methodology

### 3.1 Research framework construction

Although complex product demand analysis and design reliability research are extremely complex, complex products key components demand analysis and design reliability directly determines the overall reliability of complex products. Key components demand analysis and design reliability research are crucial to the success of complex finished product quality design. Therefore, this paper mainly focuses on the two parts of key component demand analysis and design reliability research.

**Fig 1:**
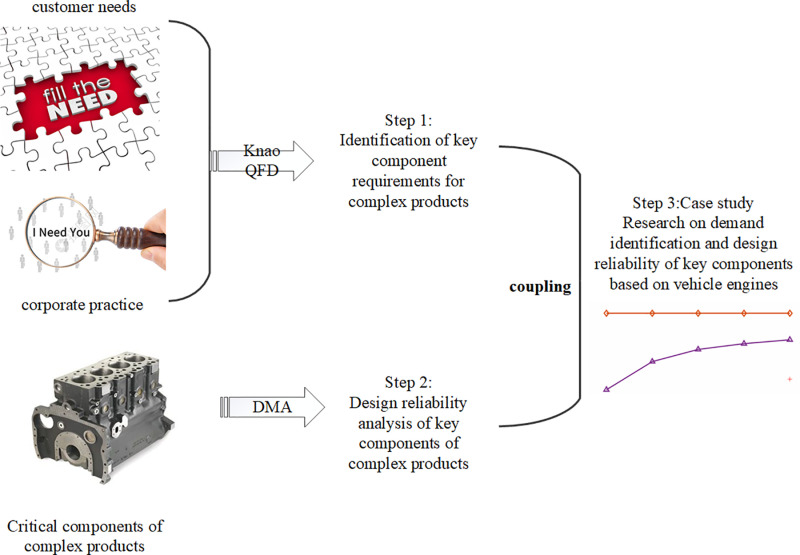
Research framework construction.

Specifically, the first part is to use Kano-QFD model to clarify the requirements of key components of complex products on the basis of fully analyzing the customer’s requirements, and to ensure that the quality characteristics of key components of complex products are consistent with the customer’s requirements. The second stage is to integrate the multi-dimensional characteristics such as product design parameters and manufacturing to construct a DMA-based reliability research method for the design of key components of complex products. Finally, the proposed method is validated by taking the demand analysis and design reliability of key components of automotive engines as an example. As shown in Fig 1.

### 3.2 Kano-QFD-based requirement analysis of critical components for complex products

#### 3.2.1 Kano model.

Professor Noriaki Kano first introduced the criteria of satisfaction and dissatisfaction into the field of quality management in 1979, and published the study Attractive Quality and Must-be Quality in 1982, which marked the maturity of Kano’s model and the theory of attractive quality.

The Kano model mainly divides requirements into the following six categories:

1) Must-be Quality (M), K<1 , usually 0.5.
2) One-dimensional Quality, (O), K=1. In general, in market research, customers talk about expected demand usually.
3) Attractive Quality (A), K>1, usually take 2. Generally speaking, Attractive Quality can bring customers an unexpected sense of satisfaction and surprise, to realize beyond the user’s expectations, and become the key to high-quality sustainable development of enterprises in the competitive market.
4) Indifferent Quality(I), K=0.
5) Reverse Demand (R), K=-1.
6) Question Quality(Q), which does not usually occur unless the question itself is problematic or improperly understood by the respondent.

Usually, the product should meet the essential needs (Must-be Quality), but for a particular product, meeting the essential needs is the most basic requirement, how to realize the one-dimensional quality? or to achieve the attractive quality is the key to the market competitiveness of complex products. Taking automobile engine as an example, to meet the must-be quality is to ensure the basic functions and performance of the engine, such as reliability, power output and fuel efficiency, etc. These must-be quality are the core requirements of automobile engine, and without these basic functions, the product will be very poor. And it is difficult for the product to be globally competitive in the market. But through continuous technological innovation and engineering improvements, the attractive performance of the engine (e.g., power output, acceleration, fuel economy, environmental protection performance, intelligent diagnostics and remote control, and silence and vibration control, etc.), which it has become the key factor for the enterprises to stand out in the competitive market and to gain the competitiveness in the market.

Generally speaking, the Kano model is usually divided into the following steps:

1) Organizing and categorizing customer needs;
2) Designing the Kano questionnaire, as shown in Table 1;
3) Counting the results;
4) Categorizing customer needs using a needs categorization assessment form.

**Table 1:** Kano questionnaire.

product	I like it very much	It should be	It doesn’t matter	I accept it reluctantly	I don’t like it very much
**Satisfy**					
**Dissatisfied**					

Organize the statistical results. If the product meets this function, it will be a matrix with five rows and one column:


$$PF_{S}\text{=}{\left\{like,shouldbe,doesn'tmatter,reluctantlyaccept,don'tlike\right\}}^{-1};$$

If the product does not fulfill this function, it is a matrix of one row and five columns:


$$PF_{DS}\text{=}\left\{like,shouldbe,doesn'tmatter,reluctantlyaccept,don'tlike\right\};$$

By multiplying the two matrices $PF_{S}*PF_{DS}$, a 5*5 matrix can be obtained. Which horizontally indicates that the product does not satisfy a certain function and need, and vertically indicates that the product satisfies certain functions and needs, as shown in Table 2.

**Table 2:** Kano model needs categorization assessment form.

product requirements		The product does not meet a need				
		I like it very much	It should be	It doesn’t matter	I accept it reluctantly	I don’t like it very much
**the product meets a need**	I like it very much	Q	A	A	A	O
	It should be	R	I	I	I	M
	It doesn’t matter	R	I	I	I	M
	I accept it reluctantly	R	I	I	I	M
	I don’t like it very much	R	R	R	R	Q

A certain requirement attribute of a product can be determined based on the calculated score of the Kano model. However, in practical applications, the requirements of complex products usually present more than one requirement, so it is necessary to determine a prioritization order for all the requirements. The frequently used method is the Better_Worse coefficient. Generally speaking, the higher the absolute value of the coefficient, the higher the demand for the product.

According to Charles et al. [38] for a specific need *i*, the satisfaction coefficient is:


$$SI_{i}=\frac{A_{i}+Q_{i}}{A_{i}+Q_{i}+C_{i}+I_{i}}$$ (1)

The satisfaction coefficient is generally positive. The user’s satisfaction will increase if the product has that demand. The larger the value, the closer it is to 1, the greater the user’s satisfaction will be.

The coefficient of dissatisfaction is:


$$DI_{i}=-\frac{C_{i}+O_{i}}{A_{i}+O_{i}+C_{i}+I_{i}}$$ (2)

The dissatisfaction coefficient is generally negative. If the product does not provide a certain need, the user’s satisfaction will decrease. The closer the value is to -1, the greater the user’s dissatisfaction.

$C_{i}$ in Eq. (1) and Eq. (2) is an adjustment parameter related to the customer satisfaction coefficient, which is the maximum value of the relative importance of demand, that is:


$$C_{i}=\max\left\{\frac{S_{i}}{\sum_{1}^{C}S_{i}},\frac{D_{i}}{\sum_{1}^{C}D_{i}}\right\}$$ (3)

$$S_{i}=\frac{A_{i}+O_{i}}{A_{i}+O_{i}+I_{i}}$$ (4)

$$D_{i}=-\frac{O_{i}}{A_{i}+O_{i}+I_{i}}$$ (5)

Combining with, the studies of Tan and Shen [39] and Tontini [40], combined with the *K* value and customer satisfaction coefficients, a new adjustment factor is proposed:


$$IR_{adj,i}={\left(1+C_{i}\right)}^{K}*IR_{o,i}$$ (6)

$$IR_{o,i}=\frac{E_{i}}{E_{o}}$$ (7)

In Eq. (6), $1+C_{i}$ is the adjustment improvement coefficient; coefficient *K* is a parameter related to the type of demand, which can distinguish the final importance of different types of customer demand. According to the characteristics of different demand types in the Kano model, the *K* is assigned a value, the must-be quality K<1, usually take the value of K=0.5, the one-dimensional quality K=1; the attractive quality K>1, usually take the value of K=2. $IR_{o,i}$ is the initial improvement coefficient, which is the ratio of the target value $E_{i}$ to the existing value $E_{o}$. Substitute the satisfaction coefficient $S_{i}$ in Eq. (4) and the dissatisfaction coefficient $D_{i}$ in Eq. (5) into Eq. (3), and optimize the initial value importance of a product demand $IR_{o,i}$ to the final importance $IR_{adj,i}$ through Eq. (7), and take this as the input of the QFD quality house matrix.

With the continuous development of new generation information technology and intelligent manufacturing, the global competitiveness of products is becoming more and more intense. Meeting customers’ attractive needs for products has increasingly become the key to product market share and corporate profitability. Therefore, in this study, attractive quality is analyzed, and the value of *K* is taken to be 2.

#### 3.2.2 QFD model.

QFD model by the Japanese Mitsubishi in the 1960s and 1970s. It is a structured method. QFD can transform customer needs into technical characteristics and successively develop complex products. And successively in the development and design of complex products, manufacturing processes, and quality and reliability management and other areas of further application. It can ensure that products meet customer needs while having high reliability, effectively reducing product failure rates, extending product life, and improving product environmental friendliness.

**Fig 2:**
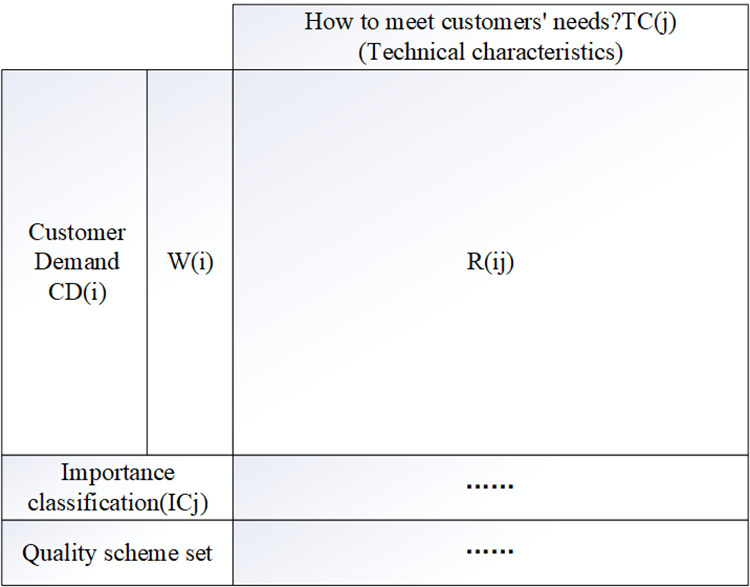
The common QFD framework diagram.

The common QFD framework diagram is shown in Fig 2 below.

QFD analysis mainly includes the following steps:

**Step1:** Identify customer demand and key technical characteristics

In the process of QFD analysis, how to accurately identify customer demand and key technical characteristics that meet customer demand is the core, through interviews, questionnaires and other ways to end consumers, product design engineers, manufacturing engineers, quality management engineers and core suppliers to carry out information collected. As shown in Fig 2, the left side of the house of quality is customer demand and importance, which are presented as row elements $CD_{i}$ and $W_{i}$, and column element $TC_{j}$ is the key technical characteristics to satisfy customer demand.

**Step2:** Basic data calculation

For any product and end customer, all quality requirements are not equally important. The Kano model is used to calculate the importance of customer demand $W_{i}$. Based on questionnaire collection from product design engineers, manufacturing engineers, quality management engineers and core suppliers, determine the quality relationship matrix $R_{ij}$, which represents the relationship value between the i−th customer demand and the j−th technical feature. Generally, relationship levels such as 1, 3, 5, 7 and 9 are used, which in turn indicate that technical characteristics and customer needs have a weak, certain, close, relatively close, and very close relationship.

**Step3:** Importance degree calculation and categorization

Calculate the importance degree of technical measure $IC_{j}=W_{i}*R_{j}$, and calculate its relative importance degree to rank the importance degree of technical measures.

**Step4:** Expanding the quality house for key components of complex products

After the establishment of the overall quality house is completed, according to the characteristics of a certain technical requirement, the technical characteristics of key components (such as hardness, roughness and performance, etc.) can also establish quality characteristics (as shown in Table 3). Through the design requirements and output characteristics of key components, the key manufacturing process can be found and the design characteristics of key components can be re-designed.

**Table 3:** Quality house of key components of complex products.

Key Component Quality Characteristics			Description of key components				
Technical requirements	Target value	Importance	Function 1	Function 2	Function 3	......	Function j
Technical requirements 1			$R_{ij}^{'}$				
Technical requirements 2							
......							
Technical requirements i							
Technology Initiative Importance Score			$IC_{j}$				

#### 3.2.3 Analysis of key component requirements of complex products based on Kano-QFD.

**Fig 3:**
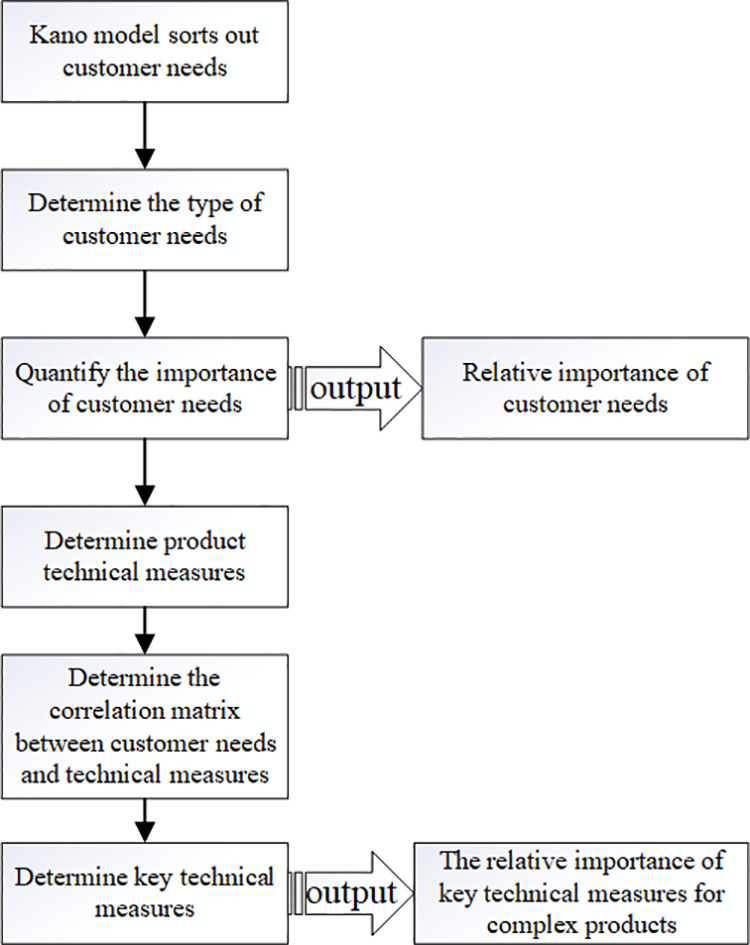
Kano-QFD-based requirement analysis process for critical components of complex products.

Combining the Kano model and the QFD analysis process forms the Kano-QFD-based requirement analysis process for key components of complex products, as shown in Fig 3.

Based on Fig 3 and the content of chapters 3.2.1 and 3.2.2, the relative importance of key technical measures for complex products can be obtained, and QFD analysis can be conducted on the technical measures of key components to obtain the breakthrough points for realizing the reliability of key components, so as to analyze complex products and Key components themselves were redesigned. However, the QFD method is mainly concerned with the conversion of user requirements into product characteristics and design parameters, but does not directly involve the reliability design aspect of products [41], nor does it particularly emphasize the consideration of reliability design and manufacturing process [42]. Therefore, while transforming customer requirements into product design functions and technical characteristics, how to consider the reliability and quality engineering management in the product design and manufacturing process has become the key to realize the reliability research of complex products.

### 3.3 Research on the reliability of design and manufacturing of key components of complex products based on DMA

For most complex products, whether the product design is reliable or not is mainly reflected in the difference between design parameter and manufacturing parameter. Design parameter are a product in a certain condition should have the attribute. Design parameters are attributes that a product should have under certain conditions. It is an ideal inherent attribute that does not change due to changes in manufacturing, internal and external environments, and human factors. For example, the strength, stiffness, adhesion, flatness, weight, thermal conductivity and design dimensions of key components. Design parameters generally exist in the form of a mathematical distribution, such as the normal distribution, Weibull distribution and so on. Taking the normal distribution as an example, it is presented with *μ* as the position parameter and *δ* as the shape parameter, and the overall shape of the curve shows a thin and high shape (e.g., for the product quality requirements of the situation is not high, it may be presented as a flat shape, but for the complex product, it is mainly presented as a thin and high shape). Manufacturing parameters are mainly reflected in the attributes of manufactured products. Affected by the manufacturing process, process quality control and upstream and downstream quality deviations, etc., they have a series of functional characteristics under specific production processes, production processes and environmental conditions. They are mainly reflected in the following Roughness, manufacturing dimensions, product hardness and functional properties, etc., are the characteristics that consumers or downstream processes require manufacturers or upstream products to provide. Typically, a product is reliable to the consumer only if the manufacturing parameters are within the product design parameters. For example, when the quality, size, roughness and strength of the product are outside the design parameters, the product is considered to be non-conforming or defective. Usually, manufacturing parameters are also in the form of a distribution, and are affected by the manufacturing process, etc., and the distribution of design parameters may not be consistent. For example, the design parameters belong to the normal distribution, but the manufacturing parameters may belong to the normal distribution, three-parameter Weibull distribution or exponential distribution, etc., especially for the early stages of complex product design and manufacturing, the distribution of its manufacturing parameters is even more uncertain.

It is assumed that the distributions of the design and manufacturing parameters both have a mean ($\mu_{d}$ and $\mu_{m}$) and a standard deviation ($\delta_{d}$ and $\delta_{m}$). If a specific event occurs in the overlapping region of the two distributions, it will lead to a failure situation in the manufactured product. For the distributions of design and manufacturing parameters, two factors are defined:

Safety margin (SM) represents the relative distance between the mean value of the design parameter and the mean value of the manufacturing parameter, which can be expressed as:


$$SM=\frac{\mu_{d}-\mu_{m}}{\sqrt[{2}]{\left(\delta_{d}^{2}+\delta_{m}^{2}\right)}}$$ (8)

Manufacturing roughness (MR) represents the standard deviation of the distribution of manufacturing parameters and can be expressed as:


$$LR=\frac{\delta_{l}}{\sqrt[{2}]{\left(\delta_{d}^{2}+\delta_{m}^{2}\right)}}$$ (9)

Among them, μ_d_ and μ_m_ are the mean values of the design and manufacturing parameters, respectively, $\delta_{d}$ and $\delta_{m}$ are the standard deviations of the design and manufacturing parameters. In fact, for most products, the design parameters and manufacturing parameters are not fixed, but exist in some statistical distribution. Therefore, for discrete and complex product manufacturing and quality and reliability management, the reliability of a critical component is the probability that the design parameter exceeds the manufacturing parameter, that is:


$$\begin{array}{l} \text{R}=P(\mu_{d}>\mu_{m})\\ =\int_{0}^{\infty }f_{\mu_{d}}(\mu_{d})[\int_{\mu_{m}}^{\infty }f_{\mu_{d}}(\mu_{d})d\mu_{d}]d\mu_{m}\\ =\int_{0}^{\infty }f_{\mu_{d}}(\mu_{d})[\int_{0}^{\mu_{d}}f_{\mu_{m}}(\mu_{m})d\mu_{m}]d\mu_{d} \end{array}$$ (10)

In the above equation, $f_{\mu_{d}}(\mu_{d})$ is the probability density function of the design parameters and $f_{\mu_{m}}(\mu_{m})$ is the probability density function of the manufacturing parameters. Assuming that $y=\mu_{d}>\mu_{m}$, where *y* is a random variable, then the above equation can be transformed into:


$$R=P(y>0)=\int_{0}^{\infty }\int_{0}^{\infty }\left(y+\mu_{m})f(\mu_{m}\right)d\mu_{m}dy$$ (11)

However, it is often the case that manufacturing parameters are influenced by multiple factors, such as whether the machine and equipment are maintenance in a timely manner, the manufacturing process, the manufacturing environment and personnel operations, etc. Due to product design defects and manufacturing processes during the manufacturing process (such as: burrs, dimensional deviations, color, hardness and strength of the product assembly process, etc.), the products produced have quality defects and cannot meet customer needs or downstream processes demand. etc. In the actual production of the enterprise, this type of data is mainly presented as the number of unqualified products produced during the production process, which is mainly discovered by manual inspection by manufacturing engineers or quality engineers or through advanced technologies such as non-destructive testing, as follows:


$$D_{r}(t)=Q_{dp}(t)/T_{o}(t)$$ (12)

In the above formula, $D_{r}$ represents the defect rate, $Q_{dp}(t)$ refers to the number of defective products in a period of time due to product design defects or manufacturing process, $T_{o}(t)$ refers to a period of time the total number of products produced (total output). $T_{o}(t)$ is the total number of products produced over a period of time.

Intelligent manufacturing equipment has a certain impact on complex products, which is reflected in equipment failures (such as mechanical failures, electrical failures, etc.), equipment aging and wear, improper equipment operation and improper equipment calibration, etc., resulting in manufacturing deviations, manufacturing interaction effects, etc. In order to measure the product failure caused by equipment on product quality and equipment interaction during the manufacturing process, this article introduces the concept of product defective rate caused by equipment.

The defective rate of products caused by equipment is mainly represented by product defects caused by equipment, which can be expressed by the following formula:


$$D_{re}(t)=Q_{e}(t)/T_{o}(t)-\varepsilon$$ (13)

In the above equation, $D_{re}$ represents the percentage of product defects or non-compliance with design parameters due to equipment failures, which can be based on product-specific defect types or quality indicators. $Q_{e}(t)$ is the number of defective products due to equipment problems over a period of time. And *ε* is a parameter that excludes the effect of the interaction between the equipment and the equipment.

Therefore, in the manufacturing process of complex products, when a product is affected by several factors simultaneously during the production and manufacturing process, its reliability can be expressed as:


$$R=\int_{0}^{\infty }f_{\mu_{d}}(\mu_{d}){\left[\int_{0}^{\mu_{d}}f_{\mu_{m}}(\mu_{m})d\mu_{m}\right]}^{n}d\mu_{d}$$ (14)

Where *n* is the number of manufacturing factors applied to the product components, where manufacturing process and equipment are the two categories of factors that have a strong influence on the manufacturing parameters [43,44].

## 4 An illustrative example

### 4.1 Background introduction

The automobile engine is the core device that provides the power source for the automobile, and it is the key component of the whole automobile that can operate normally. The performance of the engine determines the performance, reliability, safety, environmental protection and user experience of the automobile. Reliability is the ability of an engine to operate normally with expected performance under certain times and conditions without malfunction or failure. The rationality of engine design, the accuracy of the manufacturing process, the selection and quality of materials, the stability of manufacturing equipment, the quality of parts and maintenance, etc. are the key factors that affect the reliability of the engine.

The customer needs of automotive engines cover a wide range of aspects, such as: power performance, fuel economy, reliability and durability, emission control, noise and vibration, starting and driving experience, repair and maintenance costs, environmental protection and sustainability, etc. Among the above customer requirements, all of them are related to the engine block design and manufacturing process. For example: the engine block is subjected to high pressure and high temperature environments during operation, and needs to have sufficient strength and durability to ensure long-term stable operation. The engine block is an important part of the exhaust system, which has a certain impact on emission control. A well-designed block can help realize better emission control and reduce pollution to the environment. The design and production of the engine block can affect the transmission and control of noise and vibration. Appropriate block production can reduce the generation and transmission of noise and vibration, and providing a better driving experience. Easy to remove and install the cylinder block design can reduce the complexity and cost of maintenance work. The choice of materials and manufacturing processes for engine block production can have an impact on environmental protection and sustainability. Using recyclable materials or the adoption of environmentally friendly manufacturing processes can reduce the consumption of resources and the impact on the environment. Therefore, these indicators should be comprehensively considered during the engine block design and manufacturing process, and should be weighed and optimized according to needs.

Engine block is one of the core components of the engine, carrying high-pressure gas and high-temperature coolant, to measure the engine block good or bad there are a number of important indicators. Such as: Strength and stiffness, adequate strength and stiffness is to withstand the internal gas pressure and external loads, to prevent deformation and rupture of the key. Machining accuracy, engine block machining accuracy is critical to ensure a tight fit between the cylinder and piston, reducing friction losses and fuel leakage. Maintainability, the design of the engine block should take into account the convenience of repair and maintenance, including ease of disassembly and installation, convenience of replacing seals and repairing parts, etc. Lightweight, lightweight design of engine blocks is one of the current development trends, aiming to reduce the weight of the entire engine and vehicle and improve fuel economy and performance. Sealing performance, the sealing performance of the engine block is to ensure that the gas in the cylinder and the coolant does not leakage of the key. A good sealing performance helps to improve the efficiency and reliability of the engine.

### 4.2 Requirement analysis of key engine components based on Kano-QFD modeling

#### 4.2.1 Study on the importance measurement of customer requirements for automotive engines.

The development and design of automotive engine is crucial to the automotive industry. Engines with high power performance, good fuel economy, environmental friendliness, high reliability and durability of the engine, which are crucial to further improve the engine and even the vehicle market competitive advantage has a good role in promoting. However, in the process of engine design and development, many complex process information, equipment parameters and information exchange have been blocked by foreign enterprises for a long time, which makes it difficult for engine researchers and designers to convert customer needs into quantifiable and efficiently implemented product technology measures. Based on the method and improved algorithm proposed in this paper, the customer’s demand for the engine is analyzed, and the adjustment system is used to make appropriate adjustments to the customer’s demand for the engine, so that the customer’s demand can be converted into the engine technology research and development process, which further guides the engine research and development and design process.

By surveying 70 people including customers, terminal after-sales service providers, R&D engineers, manufacturing plant workers and quality management engineers, 63 pieces of valid data were finally obtained. Combined with the method of quantifying the importance of customer demand provided in this paper, the statistical results and calculation results are shown in Table 4 below, and the importance of customer demand is shown in Table 5.

**Table 4:** Statistics of engine customer requirements.

No.	Customer Demand	Frequency statistics of various types of demand						Si	Di	Ci	SI	DI	Demand Type
		A	O	M	I	R	Q						
1	Power Performance	8	11	43	1	0	0	0.950	-0.550	0.202	0.940	-0.555	A
2	Fuel Economy	12	34	14	3	0	0	0.939	-0.694	0.234	0.934	-0.695	A
3	Reliability and durability	7	45	9	1	1	0	0.981	-0.849	0.286	0.976	-0.850	A
4	Noise and Vibration	30	21	5	7	0	0	0.879	-0.362	0.187	0.876	-0.364	O
5	Repair and maintenance costs	0	5	19	36	3	0	0.122	-0.122	0.041	0.122	-0.123	M
6	Environmental friendliness	0	9	15	39	0	0	0.188	-0.188	0.063	0.187	-0.189	M
7	Innovation and technical features	17	8	19	14	5	0	0.641	-0.205	0.136	0.639	-0.208	O

**Table 5:** Importance of engine customer requirements.

Customer Demand	Classification of results	Competitive analysis			$IR_{o}$	$IR_{j}$	Sorting
		Own	opponent	Target			
Power Performance	A	8	8	10	1.250	1.806	2
Fuel Economy	A	9	8	10	1.111	1.691	4
Reliability and durability	A	9	10	10	1.111	1.837	1
Noise and Vibration	O	7	9	10	1.429	1.696	3
Repair and maintenance costs	M	7	8	10	1.429	1.458	6
Environmental friendliness	M	6	7	10	1.667	1.718	5
Innovation and technical features	O	8	7	10	1.250	1.420	7

Note: In the competitiveness analysis, 1 indicates the lowest level of satisfaction, and 10 indicates the highest level of satisfaction.

Based on the R&D engineers, after-sales personnel and market experience, using 1-10 to rate the competitiveness of the unit’s and competitors’ products. According to the content in chapter 3.2, the importance ranking of engine customer needs can be obtained. As can be seen from Table 5, reliability and durability, power performance and noise and vibration are of high concern, and need to pay extra attention and give greater resource support in the process of engine development in this unit, especially noise and vibration, this unit and the rivals are more different, and in the process of this research in the third place in the order of importance.

#### 4.2.2 Assessment of the importance of technical measures for automotive engines.

Based on the QFD content in 3.2.2, we sorted out the technical measures for reliability and durability, power performance, noise and vibration, etc. corresponding to the customer’s needs. Through exchanges with engine R&D and design personnel, manufacturing plant workers and quality management engineers, 16 technical measures were finally identified and scored and ranked in order of importance, as shown in Table 6. For example, considering the engine’s power performance, reliability and durability, environmental friendliness, noise and vibration, and fuel economy, etc., the use of high-strength steel, aluminum alloys, magnesium alloys and composites and other lightweight design technical measures in the engine design process can effectively enhance the competitiveness of the engine products.

**Table 6:** Assessment of the importance of technical engine measures.

Customer Demand	Importance	technical measures															
		A	B	C	D	E	F	G	H	I	J	K	L	M	N	O	P
Power Performance	1.806	9	7	7	9	8	5	5	7	3	3	7	5	0	0	0	7
Fuel Economy	1.691	10	7	10	7	8	7	5	7	3	0	9	3	0	3	3	9
Reliability and durability	1.837	5	7	3	7	5	3	9	10	9	3	9	10	5	9	9	5
Noise and Vibration	1.696	0	7	9	7	7	9	10	9	9	10	9	7	5	0	9	5
Repair and maintenance costs	1.458	3	7	0	3	0	5	9	9	9	3	3	0	7	9	10	0
Environmental friendliness	1.718	9	9	10	9	5	3	5	3	0	0	7	0	0	0	3	9
Innovation and technical features	1.420	7	7	9	9	9	7	7	0	0	3	9	9	7	6	3	0
Importance of technical measures		72.14	84.83	80.3	85.45	70.41	64.03	82.64	76.39	55.41	36.5	88.8	57.1	37.8	43.3	60.9	61
Ranking of importance of technical measures		7	3	5	2	8	9	4	6	13	16	1	12	15	14	11	10

Note: In Table 6, A-P represents technical measures, for example: with high-efficiency air intake system, lightweight design, with high-performance fuel systems, with variable valve timing technology, using turbocharging technology, with engine management optimization system, with precise manufacturing and assembly processes, with stringent quality control and testing, with regular maintenance and servicing instructions, with vibration and noise control technology, with advanced combustion control technology, with advanced fault diagnosis system, supports replaceable modular design, supports preventive maintenance measures, providing high-quality parts and materials, adopt advanced energy-saving technology.

As can be seen from Table 6, technical measures such as advanced combustion control technology, having variable valve timing technology, adopting lightweight design, having precise manufacturing and assembly processes, having high-performance combustion systems and having strict quality control and testing are of high importance and should be supported with resources in the engine development and manufacturing process. Advanced combustion control technology and high-performance combustion systems are positively correlated. Advanced combustion control technology and high-performance combustion systems can improve engine performance by optimizing the combustion process, fuel injection strategy and ignition timing to increase combustion efficiency and power output. Variable valve timing technology can optimize the intake and exhaust processes by adjusting the timing and magnitude of valve opening and closing to further improve engine efficiency and power performance. The lightweight design and high-quality parts and materials are positively correlated. The use of lightweight design and new materials can reduce the weight of the entire engine, improving power density and fuel economy. Precise manufacturing and assembly processes as well as strict quality control and testing can ensure that each component and system of the engine meets the design requirements, ensuring engine performance and reliability. These technical measures encompass cylinder block design and manufacturing, as well as other aspects related to engine performance and reliability, which work together to provide efficient, high performance and reliable power output throughout the engine system. Except for advanced combustion control technology and high-performance combustion systems, lightweight design and high-quality parts and materials, the correlation between technical measures is weak and not listed one by one.

After the engine assembly quality house is established based on the QFD method, the technical measures can be designed again for the secondary quality house. For example, analyze the quality requirements and technical characteristics of components such as engine cylinder bore diameter, cylinder bore position and verticality, cylinder bore cylindricity and cylinder bore surface quality. Through the establishment and analysis of the configuration-level parts quality house, deviations in the design and manufacturing process of key components can be identified, and specific measures can be given to further improve the quality and reliability of the components. Table 7 shows the engine cylinder technical quality house.

**Table 7:** Engine block technical quality house.

Key Component Quality Characteristics					Engine Block		
Technical Requirements	Target Value	Importance	Interface properties	Material selection	Wear resistance	Rigidity	Strength
surface quality	9	10	9	9	7	5	9
lightweight	7	9	7	9	5	7	7
reliability	10	10	10	9	9	9	9
price	8	8	7	7	5	7	3
manufacturing process	8	8	9	5	7	7	3
Importance			381	357	301	315	291

In order to better study the relationship between engine R&D design and manufacturing reliability, this paper takes the engine block design and manufacturing process as an example for more in-depth discussion. The engine block has the characteristics of thin-walled, porous, complex structure, high precision, complex manufacturing process and high degree of automation. In the normal operation of the engine needs to withstand long periods of high temperature, high load and intense friction and other operating environments, as well as from the cylinder head, crankshaft, oil pan, piston connecting rod assembly and other parts installed in the cylinder block load. It can be said that the cylinder block is the basis for the normal operation of the various institutions and systems on the engine, is the entire engine “skeleton”, the cylinder block is an important member of the engine.

### 4.3 Research on reliability of vehicle engine block design and manufacturing based on DMA

In the process of engine block R&D, design and manufacturing, it involves a number of important design parameters such as size, shape and surface characteristics, such as: bore diameter, bore position, bore verticality and bore surface quality. Taking the cylinder bore surface quality control and management as an example, this index measures the surface characteristics and quality of the inner wall of the cylinder bore. It mainly includes the peak height parameter, average peak height, peak and valley height parameter and stray light parameter, etc. The better the index will be more conducive to reduce friction and wear, improve sealing performance and lubrication effect and improve the surface finish and quality, etc., which directly affects the functional performance, service life and reliability of the cylinder block, therefore, it is very important to control and optimize the surface quality of the engine block. For example, Peak-to-Valley Height Parameter (Rvk) indicates the peak-to-valley height of the surface, and a smaller Rvk value indicates a smaller difference between the height of the surface, which helps to improve the sealing performance and lubrication effect. This paper analyzes the design and manufacturing process of Rvk in the actual production process as an example.

Typically, the design parameters of the cylinder bore surface quality Rvk conform to a normal distribution with a mean $\mu_{d}$ of 1.7 EA and a standard deviation $\delta_{d}$ of ± 0.5 EA. Its probability density function is:


$$f_{d}(x)=\frac{1}{\delta_{d}*\sqrt{2\pi }}*e^{\left[-\frac{1}{2}{\left(\frac{x-\mu_{d}}{\delta_{d}}\right)}^{2}\right]}=\frac{1}{0.5*\sqrt{2\pi }}*e^{\left[-\frac{1}{2}{\left(\frac{x-1.7}{0.5}\right)}^{2}\right]}$$ (15)

Data from the early stages of manufacturing complex products can provide feedback on the manufacturing process, thereby further revealing the true trends in manufacturing. By analyzing and interpreting early data, R&D and manufacturing personnel can obtain improvements related to product design, manufacturing processes, and quality control. direction. At the initial stage of new product manufacturing, the distribution of the defective rate may be affected by a variety of factors, such as equipment commissioning, process parameter adjustments, operator skills, etc. Therefore, the distribution of the defective rate can be characterized by analysis and modeling based on actual manufacturing data. Generally speaking, the manufacturing quality of new products can gradually stabilize over a period of several months. However, the exact time depends on multiple factors, including the complexity of the product, the complexity of the manufacturing process, and the effectiveness of process controls, production line stability, quality management measures, and the organization’s experience and expertise, etc. Generally speaking, the manufacturing quality of new products may have large fluctuations and instability in the initial stage. However, with the increase in production batches and the accumulation of experience, the manufacturing quality gradually becomes stable. the manufacturing quality gradually tends to be stabilized, usually, the process of manufacturing quality stabilization is generally about six months. Therefore, this article conducts analysis based on the data of the first six months of cylinder manufacturing.

**Fig 4:**
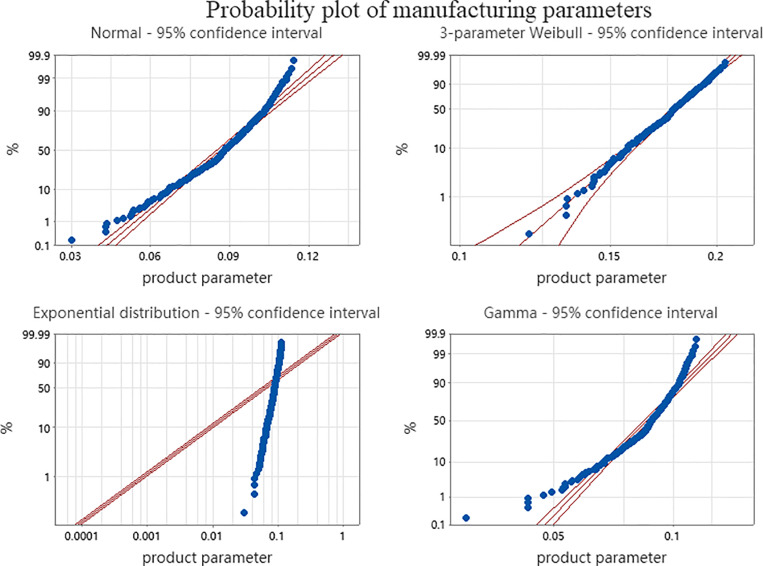
Probability plot of manufacturing data.

Taking the production process of an engine block as an example, combined with the manufacturing data of an engine manufacturing company that produced Rvk in the first six months, Minitab 17 software was used to fit the manufacturing data, and Fig 4 shows the probability plot of the manufacturing data.

Combined with Fig 4 and the goodness-of-fit test and the great likelihood estimation of the distribution parameters, etc., it can be seen that the most consistent distribution for Rvk’s manufacturing data is the three-parameter Weibull distribution, with a value of β=1.5 for the shape parameter, a value of γ=0.98 for the scale parameter and a value of η=−0.2 for the threshold parameter.

Thus, the probability density function of manufacturing parameters can be obtained as:


$$f_{m}(x)=\frac{\beta }{\eta^{\beta }}{\left(x-\gamma \right)}^{\beta -1}\exp[{\left(\frac{x-\gamma }{\eta }\right)}^{\beta }]=\frac{1.5}{{\left(-0.2\right)}^{1.5}}{\left(x-0.98\right)}^{1.5-1}\exp[{\left(\frac{x-0.98}{-0.2}\right)}^{1.5}]$$ (16)

$$R=\int_{0}^{\infty }f_{\mu_{d}}(\mu_{d}){\left[\int_{0}^{\mu_{d}}f_{\mu_{m}}(\mu_{m})d\mu_{m}\right]}^{2}d\mu_{d}$$ (17)

**Fig 5:**
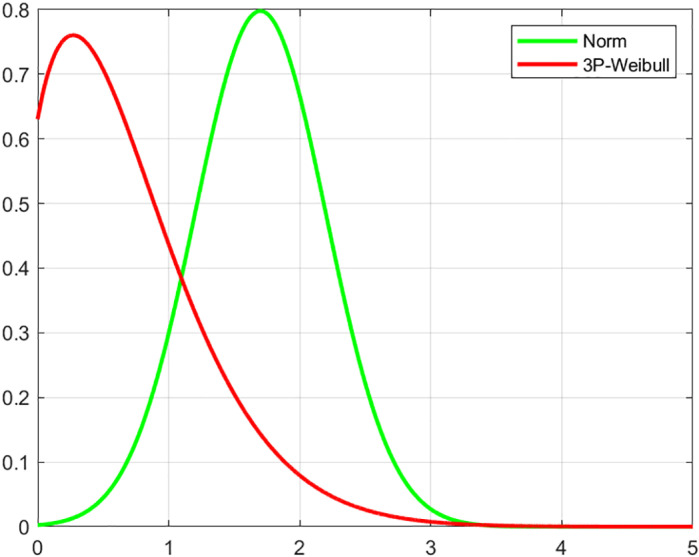
Design parameter and manufacturing parameter probability density curve.

For the case where the design parameters conform to normal distribution and the manufacturing parameters conform to three-parameter Weibull distribution due to two-factor influence, the integral of the above equation is relatively difficult to solve, so MATLAB is used to summarize the two distribution functions in the same graph respectively, as shown in Fig 5 shown.

From Fig 5, it can be seen that the manufacturing parameters before the intersection point are larger than the design parameters, and at this time the product is a failure state, that is, the product produced before the intersection point is a failure state. Because the two distributions are not both normal, it is not possible to find their closed-form solutions. Therefore, Monte Carlo simulation is used to solve it.

**Fig 6:**
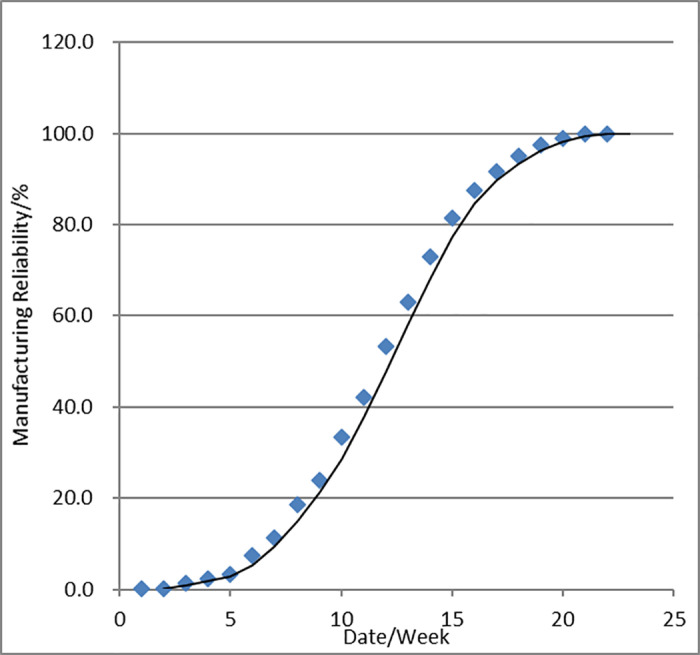
Engine block manufacturing reliability.

From Fig 6 can be seen in the manufacturing cycle to 20 weeks, the manufacturing reliability reaches 98.8%, the follow-up will be steadily above 99%. According to the company’s actual production rhythm (45JPH, 8 hours/day), the enterprise daily average due to equipment, manufacturing process and other reasons for the production of defective products is about 43 pieces (45*8*0.12=43.2), and the data is also from the enterprise actual research data The data is not far from the actual research data from the enterprise (the midpoint in Fig 6 represents the actual company, and the curve represents the curve fitted in this article). It can be seen that the research in this paper for the guidance of the enterprise to rationalize the development of the process, the maintenance of production equipment has a certain practical significance.

## 5 Conclusions and future research

In the study of complex product design reliability, it is necessary to identify the quality characteristics of key components of complex products with the help of users, manufacturing personnel, quality management engineers and operation and maintenance data, and considering the design and manufacturing reliability of key components at the early stage of design has become an important part of the study to evaluate the reliability of complex product design.

In this paper, combining market-oriented demand analysis and engineering-oriented quality improvement, the Kano-QFD model is used to clarify the path of identifying the quality characteristics of the key components of complex products, and ensuring that the quality characteristics of key components of complex products are consistent with customer needs, so as to improve the overall reliability of complex products.

On the basis of demand analysis, integrating multi-dimensional characteristics such as product design parameters and manufacturing, a DMA-based reliability research method for the design of key components of complex products is constructed. It provides a new research direction for the early reliability research of complex product design, and is verified by taking the engine cylinder as an example. The results show that the method proposed in this article is consistent with the actual situation, and further illustrates the effectiveness of the method proposed in this article.

Finally, taking the engine as an example, through the research on engineering designers, end customers, quality management personnel, manufacturing management personnel and other personnel, and by adopting Kano-QFD and other methods for demand identification and quality characteristics of key components. The results show that: the cylinder block plays an important role in engine performance, reliability and fuel economy; the design and manufacturing reliability of the cylinder block in the early stage of design were studied through the DMA method, further revealing the true trend of manufacturing, and through analysis and interpret preliminary data to provide R&D and manufacturing personnel with improvement directions related to product design, manufacturing processes, and quality control.

The development cycle of complex products is long, the process is complex, there are numerous uncertain factors, and the reliability data presents various forms of expression, multiple sources, and low value density. At the same time, due to the coupling effect between manufacturing and equipment, the reliability of manufactured products is often lower than the design target. In the future, we will further explore the following aspects of research: 1) Use industrial software and historical data to simulate the initial design reliability of new products to reduce waste in the actual production process, for example, improving data collection methods, exploring the coupling relationship between design and manufacturing, adopting super powerful industrial software, etc. 2) Production equipment, environment, and human factors engineering will all have a significant impact on design and manufacturing. So, further study the quality coupling effect between equipment and products in the production process, especially in distinguishing between equipment manufacturing issues and product design issues, which is crucial for future reliability analysis of complex product DMA.
